# A high-efficiency PEG-Ca^2+^-mediated transient transformation system for broccoli protoplasts

**DOI:** 10.3389/fpls.2022.1081321

**Published:** 2022-12-12

**Authors:** Dongxu Yang, Yongyu Zhao, Yumei Liu, Fengqing Han, Zhansheng Li

**Affiliations:** Key Laboratory of Biology and Genetic Improvement of Horticultural Crops, Ministry of Agriculture, Institute of Vegetables and Flowers, Chinese Academy of Agricultural Sciences, Beijing, China

**Keywords:** broccoli, protoplast, gene function, transient transfection, subcellular localization

## Abstract

Transient transformation of plant protoplasts is an important method for studying gene function, subcellular localization and plant morphological development. In this study, an efficient transient transformation system was established by optimizing the plasmid concentration, PEG4000 mass concentration and genotype selection, key factors that affect transformation efficiency. Meanwhile, an efficient and universal broccoli protoplast isolation system was established. Using 0.5% (w/v) cellulase R-10 and 0.1% (w/v) pectolyase Y-23 to hydrolyze broccoli cotyledons of three different genotypes for 3 h, the yield was more than 5×10^6^/mL/g, and the viability was more than 95%, sufficient to meet the high standards for protoplasts to be used in various experiments. The average transformation efficiency of the two plasmid vectors PHG-eGFP and CP507-YFP in broccoli B1 protoplasts were 61.4% and 41.7%, respectively. Using this system, we successfully performed subcellular localization of the products of three target genes (the clubroot resistance gene *CRa* and two key genes regulated by glucosinolates, *Bol029100* and *Bol031350*).The results showed that the products of all three genes were localized in the nucleus. The high-efficiency transient transformation system for broccoli protoplasts constructed in this study makes it possible to reliably acquire high-viability protoplasts in high yield. This research provides important technical support for international frontier research fields such as single-cell sequencing, spatial transcriptomics, plant somatic hybridization, gene function analysis and subcellular localization.

## Introduction

Broccoli (*Brassica oleracea* L. var. *italica*) is a variety of cruciferous cabbage known as the “ nutritional powerhouse” because of its rich content of nutrients such as protein, vitamin C and minerals (Gasper et al., 2005; Fahey et al., 2019; Li et al., 2022). Recent studies have found that broccoli is also rich in sulforaphane (Li Z et al., 2021; Li et al., 2022), an active anticancer compound that significantly reduces the occurrence of various cancers, including liver, lung, stomach, breast, bladder, colorectal, and prostate cancers (Keck et al., 2003; Chilakala et al., 2020; Mahn and Castillo, 2021; Li et al., 2022; Xie et al., 2022). It’s reported that sulforaphane could prevent and reduce the risks of cardiovascular and cerebrovascular diseases, hypertension, myopia, depression syndrome, and other conditions (Georgikou et al., 2020). At present, according to the information center of the Ministry of Agriculture, the area in which broccoli is sown in China exceeds 120,000 hectares, accounting for more than 20% of the global output, and China is the main exporter of broccoli (Li et al.,; Li et al., 2019; Huang et al., 2021).

*Agrobacterium*-mediated genetic transformation technology has been widely used in cruciferous crops, such as oilseed rape (*Brassica napus*) (Bates et al., 2017), mustard (*Brassica juncea* L. Czern.) (Dutta et al., 2008), cabbage (*Brassica oleracea* var. *capitata*) (Brown and Wang, 2004), broccoli (Chen et al., 2001), and Chinese cabbage (*Brassica campestris* L. ssp. *pekinensis*) (Min et al., 2007), but this method is laborious and yields low transformation efficiency. In addition, there is severe genotype dependence, and self-pollination is required to obtain the T1 generation to observe phenotypes or perform component identification, which increases the time required (Gould et al., 1991; Radke et al., 1992; Wang et al., 2015; Wang Y et al., 2022). Therefore, development of a fast, efficient and genotype-independent genetic transformation system that can speed up the gene function verification of important traits is of great significance. Such a system would also play a significant role in improving the efficiency of mining beneficial genes from the reported genomes of *Brassica* cops. At present, no such system has been reported for important vegetable crops of the *Brassica* genus of the family Cruciferae.

Transient transformation of protoplasts can be used to quickly transfer a large number of target genes for expression and verification in a short period of time (Abel and Theologis, 1994; Duarte et al., 2016). There are many methods for transient transformation, including particle bombardment, microinjection, PEG-mediated transfection, lipofection-mediated transfection and electronic transfection (Wang Y et al., 2022). Among these methods, PEG-mediated transient transformation has the advantages of simple operation, strong universality and stable transformation efficiency (Liu and Friesen, 2012; Sahab et al., 2019a; Wang Y et al., 2022). The establishment of a high-efficiency transient transformation system for broccoli protoplasts, is of great importance for research on gene function, plant physiological and biochemical processes, molecular mechanisms, and some protoplast-based research (single cell, spatial transcriptomics and somatic hybridization) in the cruciferous crops.

In this study, we constructed a high-yielding and high-viability protoplast isolation and purification system for broccoli. Then we took the lead in establishing an efficient and stable transient transformation system for broccoli protoplasts that can be used for rapid analysis and validation of gene functions. Based on this system, we successfully determined the subcellular localization of the gene product of the enhanced rhizoctonia-resistance gene *CRa* (Ueno et al., 2012) of *Brassica* and those of two key glucosinolate regulatory genes, *Bol029100* and *Bol031350* (Rubel et al., 2020).

## Results

### Isolation of protoplasts from broccoli cotyledons

**Figure 1:**
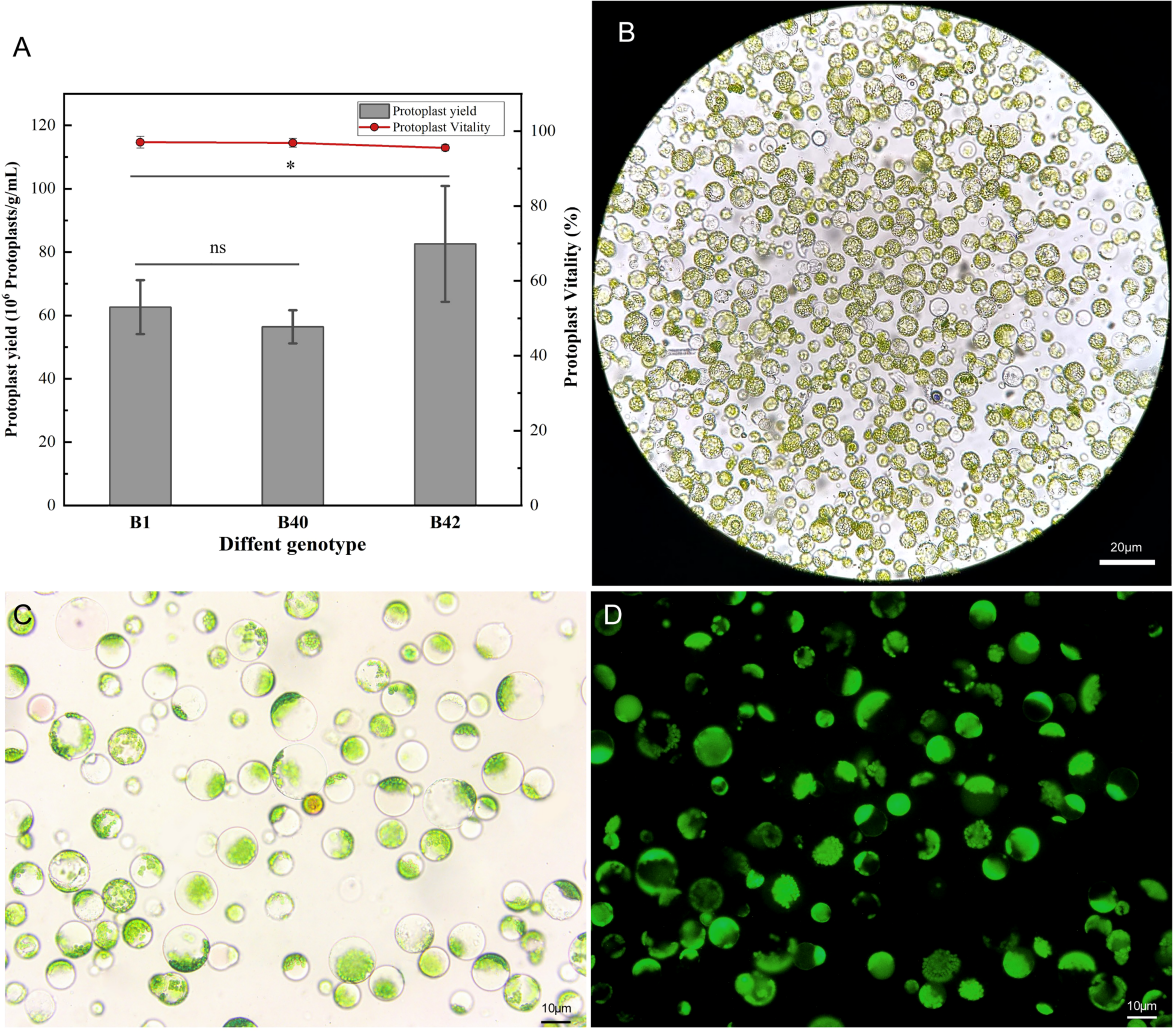
Yield and viability of cotyledon protoplasts of different broccoli genotypes. **(A)** Statistics on cotyledon protoplast yield and viability of three genotypes of broccoli, B1, B40 and B42. Data represent the means of 3 values, and error bars show the standard error values; Statistical significance levels (Student’s t-test) are shown: ns, no significant; *p < 0.05. **(B)** Microscopic examination of B1 genotype protoplast, scale bar = 20 μm. **(C)** The bright field images of B1 genotype protoplast for fluorescein diacetate (FDA) microplate assay, scale bar = 10 μm. **(D)** The dark field images of B1 genotype protoplast for fluorescein diacetate (FDA) microplate assay, scale bar = 10 μm.

A high-efficiency protoplast isolation system was successfully constructed using broccoli explants of various genotypes. To determine the universality and efficiency of the system, three genotypes of broccoli protoplasts were all isolated by the same method. The cotyledons of sterile seedlings that had been cultured for 7-10 days were preincubated in CM solution (**Table S1**) for 12 h, and strips of approximately 1 mm were then cut from the leaves and placed in 0.3 M mannitol enzymatic hydrolysis solution for enzymatic hydrolysis. The process did not require a vacuum environment and involve toxic reagents, which indicated it was simple and reliable in operation. The leaf material was hydrolyzed with 0.5% (w/v) cellulase R-10 and 0.1% (w/v) pectolyase Y-23 on a shaking table for 3 h. Using this method, the yield of B1, B40, and B42 protoplasts all reached or exceeded 5.05×10^6^ protoplasts/g. The B1 genotype yielded 5.33×10^6^-7.05×10^6^ protoplasts/g, the B42 genotype yielded 6.23×10^6^-9.84×10^6^ protoplasts/g, and the yield from the B40 genotype was 5.05×10^6^-6.04×10^6^ protoplasts/g. These yields met the standard needed for protoplast transformation experiments (1.0×10^6^-2.0×10^6^ protoplasts/mL). At the same time, the protoplast viability was determined by fluorescein diacetate (FDA) to be greater than 95% (**Figure 1**) (**Table S2**). Microscope observation showed that the purified protoplasts were relatively unfragmented and that the preparations contained little cell debris. Thus, they fully met the standard required for various protoplast-related experiments. From this result, protoplasts with high viability were obtained in high yield from the cotyledons of broccoli plants of the three genotypes, and there were no significant differences between the B1 with the B40 in yield or viability (*p* < 0.05), demonstrating the universality and efficiency of the method.

### Construction of a transient transformation system for broccoli protoplasts

**Figure 2:**
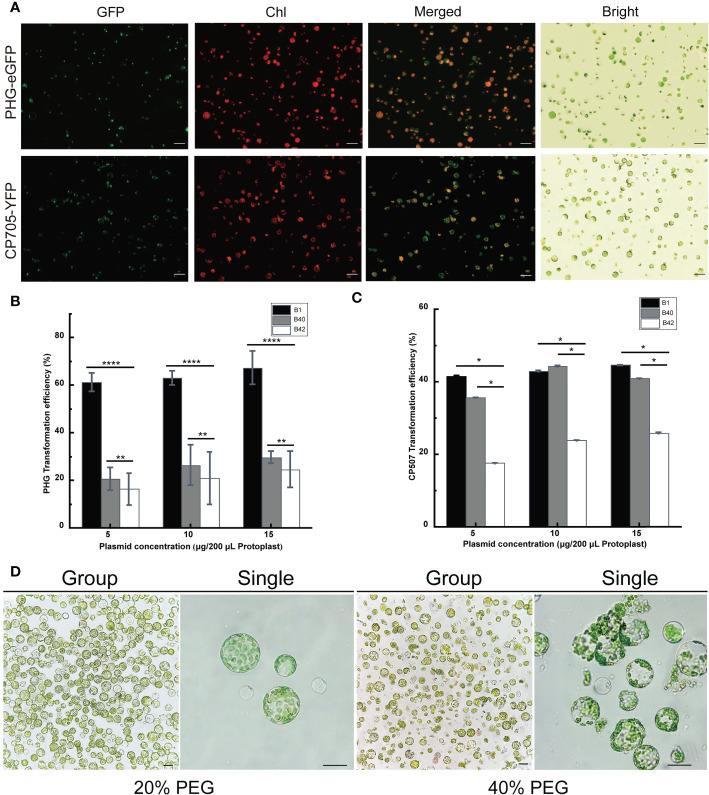
Optimization of the transient transformation system for broccoli protoplasts. Transient expression of different constructs in B1 protoplasts. Samples were visualized under a fluorescence microscope. **(A)** The 35S::eGFP PHG-eGFP and 35S::YFP CP507-YFP were transiently expressed in B1 cotyledon protoplasts. Merged images of GFP or YFP and chlorophyll autofluorescence (Chl) as well as bright field images of protoplasts were shown. Scale bar = 20 μm. **(B, C)** Comparison of transfection efficiency of 5-15 μg/200 μL protoplasts PHG-eGFP and CP507-YFP plasmids in B1, B40 and B42 cotyledon protoplasts, expressed as the ratio of GFP-positive cells to the total number of protoplasts (n≥100). Data represent the means of 3 values, and error bars show the standard error values; Statistical significance levels (Student’s t-test) are shown: ns, no significant; *p < 0.05; **p < 0.01; ****p < 0. 0001. **(D)** After 20% PEG4000 and 40% PEG4000 induction treatment for 20 min, the protoplasts were incubated in W5 solution for 12 h to observe the protoplast culture by microscopy. Group images and single images were observed under 10× and 40× microscope, respectively. Scale bar = 10 μm.

The PHG-eGFP (12.7 kb) and CP507-YFP (10.7 kb) plasmids were transferred into the prepared B1 protoplasts, and the protoplasts were cultured at 25°C for 13-16 h in the dark. The green fluorescence signal of GFP in protoplasts transfected with PHG-eGFP and the yellow fluorescence of YFP in protoplasts transfected with CP507-YFP were observed under excitation at 488 nm (**Figure 2A**). The result showed that the plasmid DNA that encoded the fluorescent protein could be transferred into broccoli protoplasts and the fluorescent proteins were stably expressed. The two plasmids were also transformed into B40 and B42 protoplasts, and the fluorescent proteins were also stably expressed in those protoplasts. Thus, a transient transformation system for broccoli protoplasts was successfully constructed in this study.

### Effect of plasmid concentration on protoplast transformation efficiency

A total of 5 μg, 10 μg, and 15 µg plasmid vectors of PHG-eGFP and CP507-YFP were transferred into 200 μL (approximately 4 × 10^5^) protoplasts obtained from broccoli genotypes, and the resulting transformation efficiency was calculated shown in **Figures 2B, C**. Although the average efficiency of transformation by PHG-eGFP and CP507-YFP increased slightly as the plasmid concentration increased, there was no significant difference between treatments in any of the three genotypes (*p* < 0.05). When the plasmid mass was increased stepwise from 5 μg to 15 μg, the efficiency of transformation of B1 protoplasts by PHG-eGFP was respectively 58.6-65.8%, 59.5-64.7%, and 59.4-73.1%, with mean values of 61.4%, 63.0%, and 67.4%, respectively. For B40, they were respectively 15.5-25.0%, 17.6-34.5%, and 27.4-32.4%with mean values of 20.6%, 26.6%, and 29.7%. And in B42, they were 12.8-24.5%, 10.4-32.2%, and 16.8-30.4% with mean values of 16.3%, 21.4%, and 24.7%, respectively. When the same amounts of the CP507-YFP plasmid were used, the efficiency of transformation of B1 were respectively 42.2-44.8%, 30.0-55.2%, and 38.2-48.8% with mean values of 41.7%, 43.0%, and 44.7%; the efficiency for B40 were respectively 25.4-41.8%, 35.0-53.0%, and 38.8%-44.2% with mean values of 35.7%, 44.4%, and 41.0%. While the efficiencies for B42 were respectively 15.4-19.8%, 21.2-27.4%, and 20.0-33.3% with mean values of 17.7%, 24.0%, and 26.0%. The results indicated that over the range of 5-15 μg plasmid/4×10^5^ protoplasts, the amount of plasmid had little or nearly no effect on the transfection efficiency. The transformation efficiency of PHG-eGFP in B1 varied the most with increasing plasmid concentration (58.6%-73.1%). The average transformation efficiency of the other two plasmids were less than 9.1% (**Figures 2B, C**) (**Table S3**).

### Effect of PEG4000 mass concentration on cotyledon protoplast transformation efficiency

Totally, 5 μg PHG-eGFP vectors were added to 200 μL of B1, B40 and B42 cotyledon protoplasts, and transfection was induced by the addition of an equal volume of 20% (w/v) PEG4000 or 40% (w/v) PEG4000 PEG-Ca^2+^ and incubation for 20 min. The results showed that the efficiency of transformation using 40% PEG4000 was higher than 20% PEG4000, but when the protoplasts were cultured in the presence of 40% PEG4000, a large number of the protoplasts ruptured, and this seriously affected the subsequent observation and culture of the protoplasts (**Figure 2D**). Similarly, the protoplasts transformed by CP507-YFP in the presence of 40% PEG-Ca^2+^ showed obvious rupture. Thus, this study found that the optimal mass concentration of PEG4000 for cotyledon protoplast transformation of the three broccoli genotypes was 20%.

### Transformation efficiency of different broccoli genotypes

The transformation efficiency of protoplasts for B1, B40 and B42 genotypes were compared under the conditions of 5 μg plasmid concentration and 20% PEG-Ca^2+^ induction solution. From **Figure 2A** and **Table S3**, we could find that the PHG-eGFP and CP507-YFP plasmid vectors all yielded higher transformation efficiency in B1 protoplasts, and that there were significant differences in the transformation efficiency obtained using different plasmid vectors (*p* < 0.05) to 65.8% with an average of 61.4%. And for CP507-YFP, transformation efficiency ranged from 38.2% to 45.0% with an average of 41.7%. The transformation efficiency of PHG-eGFP and CP507-YFP were both in the lowest level in B42 protoplasts, which respectively ranged from 12.5% to 24.8% and from 15.0% to 20.6% with the average values were 16.3% and 17.7%. So, our research had shown that the efficiency of protoplast transformation was obviously influenced by recipient genotypes and by vector types.

### Subcellular localization of target genes

**Figure 3:**
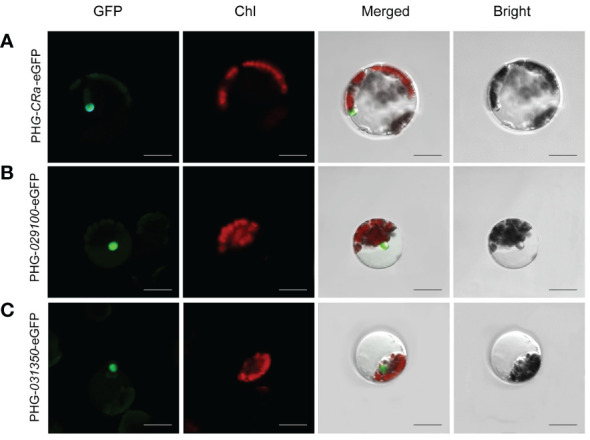
Subcellular localization of *CRa*, *Bol021900* and *Bol031350* genes. **(A-C)** presented PHG-*CRa*-eGFP, PHG-*029100*-eGFP and PHG-*031350*-eGFP were all located in the nucleus and transiently co-expressed in broccoli B1 protoplasts; Individual and merged images of GFP and chlorophyll autofluorescence (Chl) as well as bright field images of protoplasts were shown. Scale bars = 10 μm.

The expression of three target genes, *CRa*, *Bol021900* and *Bol031350*, linked to GFP in B1 protoplasts cultured in W5 suspension was verified by laser confocal microscopy. It has been reported that *Bol021900* and *Bol031350* are involved in the regulation of sulforaphane metabolism and that *CRa* expression significantly enhances resistance to clubroot in *Brassica* crops (Ueno et al., 2012; Ce et al., 2021). After induction with 20% PEG-Ca^2+^, the protoplasts were cultured in W5 solution for 12-15 h. Under microscopic observation, green fluorescence was observed in the protoplasts, and the three gene products and their fusion proteins were all located in the nucleus (**Figure 3**). The expression of fluorescent proteins in broccoli protoplasts demonstrated the validity and stability of this transformation system, which showed that it can be successfully applied to study the subcellular localizations of the products of specific genes.

## Discussion

To date, protoplasts have been successfully isolated from approximately 40 families, more than 100 genera, and more than 400 species of plants (Reed and Bargmann, 2021). The protoplasts have been successfully isolated from the family Cruciferae focus on *Arabidopsis. thaliana (* Yoo et al., 2007; Ryu et al., 2019), and there are a few reports on *Brassica napus (* Kang et al., 2017; Li X et al., 2021), Chinese cabbage (Sivanandhan et al., 2021), cabbage (Fu et al., 1985) and cauliflower(*Brassica*. *oleracea* var. *botrytis*) *(* Yang et al., 1994), but some common problems such as low protoplast yield and viability still be improved. Despite the fact that there are a few reports on transient transformation systems based on protoplasts in Cruciferae, the problems of low transformation efficiency and genotype dependence restrict the wide application of this technology (Sun et al., 2019; Sahab et al., 2019b). At present, plant protoplast-based technologies are being widely used in frontier fields such as single-cell sequencing (Armand et al., 2021; Marand et al., 2021), subcellular localization (Mackon et al., 2021), somatic cell hybridization (Xia, 2009), and plant morphogenesis (Yu et al., 2020). The establishment of an efficient and universal protoplast isolation system is the experimental premise and the necessary support for research in the above frontier fields.

In this study, a genotype-independent high-efficiency protoplast isolation and purification technology system for broccoli was successfully constructed. The yield and vitality of protoplasts obtained by this method are sufficient to meet the needs for transformation and various related experiments based on protoplasts. The coordination of enzymatic hydrolysis time with enzyme hydrolysate concentration ratio is a key factor in obtaining high protoplast yield and high viability (Liqing et al., 2005; Cao et al., 2014; Beard et al., 2021). In this study, 0.5% cellulase and 0.1% pectinase were used to isolate protoplasts from the cotyledons of broccoli of three different genotypes; the use of an enzymatic hydrolysis time of 3 h ensured high yield (> 5×10^6^/g) and high vitality (> 95%) of the resulting protoplasts. The broccoli protoplast isolation and purification system constructed in this study is superior to the systems previously reported for other cruciferous crops such as Chinese cabbage (Sivanandhan et al., 2021), cabbage (Kiełkowska and Adamus, 2019), rape (Li X et al., 2021), cauliflower (Yang et al., 1994) and others. In addition, we observed that broccoli genotype is not a key factor affecting protoplast yield and viability. Similar protoplast yields and viabilities were obtained for the three tested genotypes of broccoli under the same enzymatic hydrolysis conditions, indicating that the system is universal to broccoli materials of different genotypes. This finding provides an important scientific basis for improving the yield and viability of protoplasts isolated from other cruciferous crops.

Transient transformation system for protoplast mediated by PEG-Ca^2+^ have been successfully constructed and applied in field crops such as maize(*Zea mays* L.) (Cao et al., 2014; Hu et al., 2020), wheat(*Triticum aestivum* L.) (Luo et al., 2022), rice(*Oryza sativa* L.) (Page et al., 2019) and cotton(*Gossypium* spp) (Wang P et al., 2022), but the use of such systems in cruciferous crops has been limited to *A. thaliana*. There are several reports on cabbage and Chinese cabbage, but the transformation efficiency of those two species is less than 45%, and the transient transformation system displays serious genotype-dependent performance (Sun et al., 2019; Sahab et al., 2019b). The PEG-Ca^2+^-mediated protoplast transient transformation system established in this study was also genotype-dependent, and the transformation efficiency of the three broccoli genotypes differed significantly (*p* < 0.05). The transformation efficiency of the three plasmids in the inbred broccoli line of B1 genotype varied widely from 39.7.6% to 89.2%, and the efficiency was only 15.5% to 53.8% in the B40 genotype and 10.9% to 45.2% in the B42 genotype. It verifies that genotype is an important factor that affects the instantaneous transformation efficiency of *Brassica* crops such as broccoli (Sun et al., 2019; Sahab et al., 2019b). At the same time, our study found that a higher transient transformation efficiency can be obtained in broccoli genotype B1 mediated by *Agrobacterium*, which suggested that genotype good for genetic transformation can ensure a rapid acquisition of its genetically transformed progeny (Han et al., 2021).

We also optimized two other key factors that affect broccoli protoplast transformation: plasmid concentration and mass concentration of PEG4000. In this study, we found no significant difference (*p* < 0.05) in transformation efficiency in the same broccoli genotype at plasmid concentrations of 5-15 μg, consistent with the results of previous studies (Zhang et al., 2011). The optimal mass concentration of PEG4000 for inducing transient transformation of broccoli protoplasts was 20%, not the 40% PEG4000 concentration that is used in most plant protoplasts (Yoo et al., 2007; Zhang et al., 2011; Cao et al., 2014; Biswas et al., 2022). At a PEG4000 concentration of 40%, the transformation efficiency of broccoli protoplasts was somewhat improved, but protoplast rupture was observed after culture of the material; this is a novel finding. In conclusion, genotype and the specific plasmid used both had significant effects on the efficiency of transformation of cyanobacterial protoplasts. Thus, to improve protoplast transformation efficiency, we should first select genotypic materials that have the potential for high transformation efficiency and then choose an appropriate PEG4000 induction concentration. The mechanism by which genotype affects the transformation efficiency of protoplasts remains to be further investigated.

Finally, we studied the subcellular localization of three target genes, *Bol029100*, *Bol031350* and *CRa*. And the *Bol029100* and *Bol031350* genes encode FMO _GS- OX5_ (flavin-containing monooxygenase, FMO _GS-OX5_); broccoli contains two homologous copies of this gene (Wang et al., 2011; Zhao et al., 2021), which is present in only one copy in *Arabidopsis* (*AT1G12140*) (Kong et al., 2016). The gene encodes flavin monooxygenase (FMO _GS-OX_), which catalyzes the oxidation of sulfur atoms in methylthioalkyl mustard oleosides (MT GSLs) to produce the anticancer active precursor of sulforaphane (SFN, SF), 4-methyl sulfinyl butylthioside (glucoraphanin, GRA) (Lee et al., 2017; Wang et al., 2017; Tao et al., 2022). While we found that two homologous copies of the gene are present in broccoli, and the functional differences between these genes need to be further verified. This study provides a scientific basis for revealing their functional properties and investigating their expression. Subcellular localization of the protein encoded by the clubroot resistance gene *CRa*, was firstly reported in broccoli, which might provide more evidence of clubroot resistant in *Brassica oleracea* crops.

**Figure 4:**
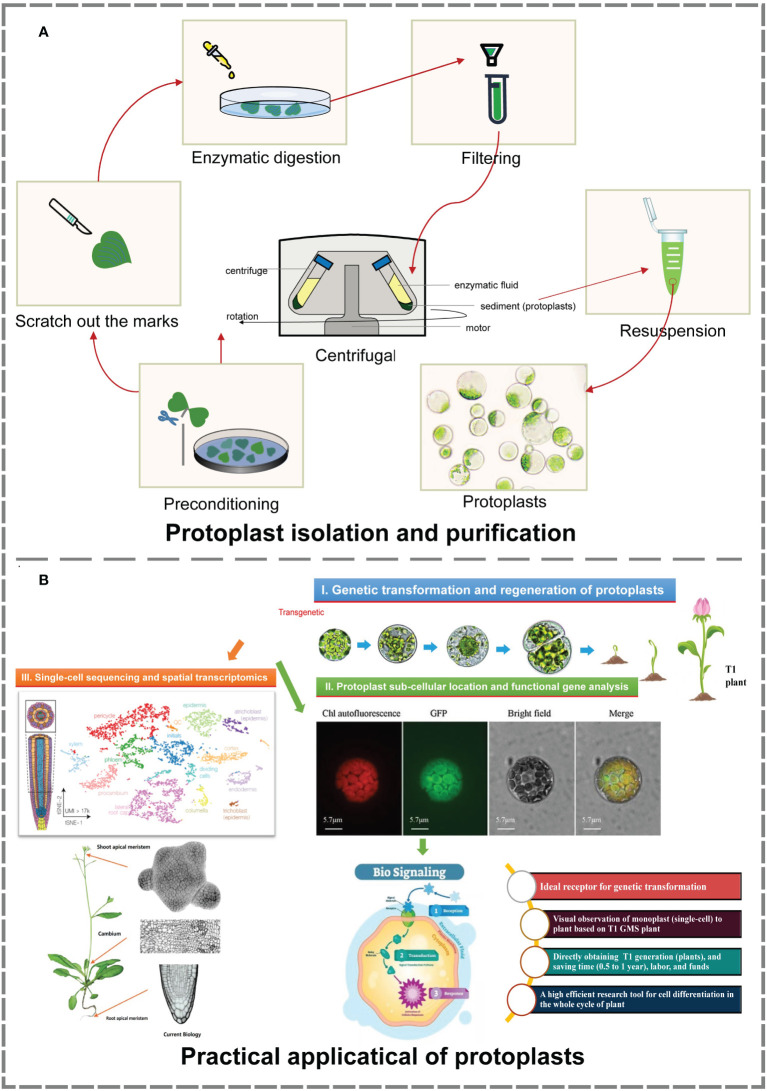
Isolation, purification and practical application of protoplasts (Buckley, 2015; Greb and Lohmann, 2016; Tanveer and Yousaf, 2020; Moses and Pachter, 2022). **(A)** The process of protoplast isolation and purification. The material and production of images based on our research. **(B)** Main practical applications of protoplasts. Image material from references, typesetting and production based on our research.

In this study, an efficient isolation and transient transformation system for broccoli protoplasts were constructed. This system makes possible the acquisition of high-yield and high-viability protoplasts and provides important technical support for research in frontier fields such as single-cell sequencing, spatial transcription analysis, somatic cell hybridization, gene function analysis, and subcellular localization. Using this transformation system, T1-generation transformants can be directly obtained, facilitating the observation and analysis of their development from single cells to plants. Moreover, this system reduces the culture time needed to obtain positive plants, greatly enhancing the transformation efficiency. Above all, it provides new ways and new ideas for histological analysis, gene function verification and morphological construction (**Figure 4**).

## Conclusions

We constructed an efficient protoplast isolation and purification system for broccoli that is universal for different genotypes and can be used to obtain protoplasts with high viability in high yield, thus overcoming the limitation of genotype dependence. A PEG-Ca^2+^-mediated efficient transient transformation technology system for broccoli protoplasts was optimized and established. The transformation efficiency it yielded was significantly higher than those provided by systems previously developed for cruciferous crops and showed significant enhancement compared with systems for non-cruciferous crops. A new finding of this study is that the use of 20% PEG4000 during induction yielded significantly better than 40% PEG4000 in broccoli, which better ensured the integrity of the resulting protoplasts. In summary, the efficient transient transformation system for broccoli protoplasts can be successfully applied to subcellular localization studies. The system provides a new method and technical support for protoplast isolation, subcellular localization studies and research on gene function, plant physiological and biochemical processes, molecular mechanisms in cruciferous crops such as broccoli.

## Materials and methods

### Materials and reagents

**Figure 5:**
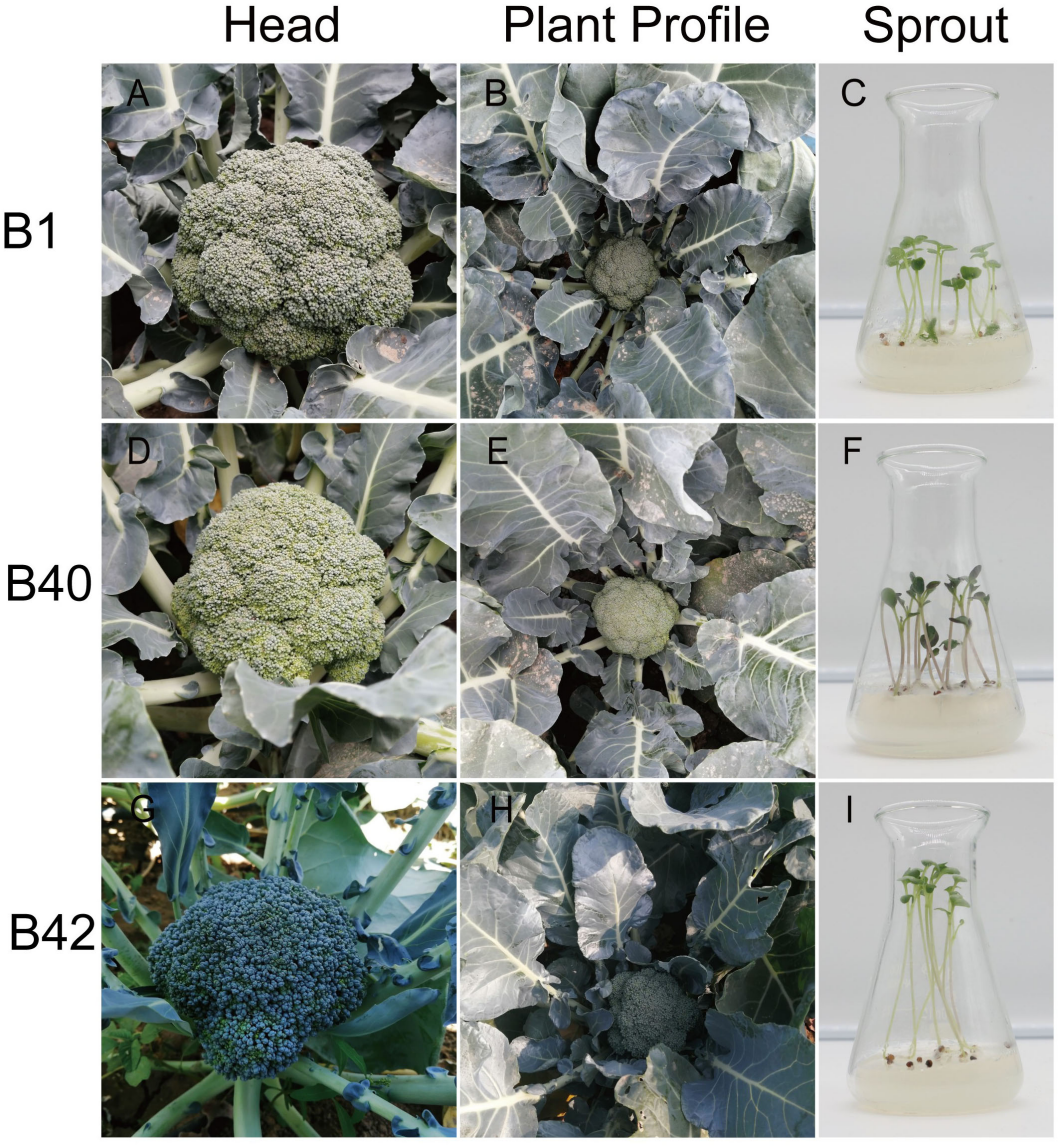
The profiles of broccoli plants and sprouts. **(A–C)** Head and plant profiles of B1 genotype broccoli plant. Representative healthy 10-day-old B1 broccoli sprout on seeding medium used for protoplast isolation. **(D–F)** Head and plant profiles of B40 genotype broccoli plant. Representative healthy 10-day-old B40 broccoli sprout on seeding medium used for protoplast isolation. **(G–I)** Head and plant profiles image of B42 genotype broccoli plant. Representative healthy 10-day-old B42 broccoli sprout on seeding medium used for protoplast isolation.

The cotyledons of sterile seedlings of various genotypes of the broccoli inbred lines B1, B40 and B42 were used as exosomes for protoplast isolation (**Figure 5**). All materials were cultivated by Institute of Vegetable and Flower Research, Chinese Academy of Agricultural Sciences (IVF-CAAS). B1 was the F_6_ generation of the inbred line material, and it exhibited medium-early maturity, semierect plant type, dome-shaped head, blue−green buds, medium fine flower buds, and no lateral branching in the field (**Figure 5A**). B40 was the F_6_ generation of the inbred line material, and it showed medium-late maturity, erect plant type, semidome-shaped head, dark green buds, medium flower buds and no lateral branching in the field (**Figure 5B**). B42, the F_7_ generation of inbred line material, exhibited early maturity, semierect plant shape, dome-shaped head, green bulbs, fine buds and few lateral branches in the field (**Figure 5C**).

**Figure 6:**
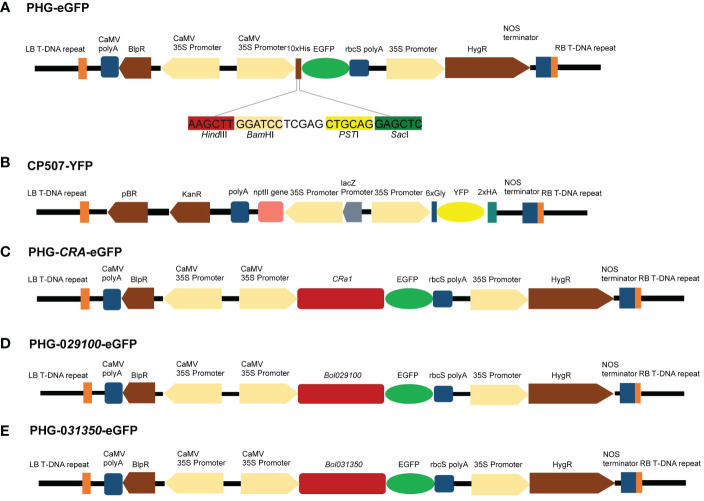
Maps of vectors with target genes. **(A)** Genetic map of PHG-eGFP and information of four enzymatic cut sites. **(B)** Genetic map of CP507-YFP. **(C)** Genetic map of PHG-*CRa*-eGFP. **(D)** Genetic map of PHG-*029100*-eGFP. **(E)** Genetic map of PHG-*031350*-eGFP.

An eGFP expression cassette was created using the plasmids PHG-eGFP (12.7 kb), which encode green fluorescent protein and promote eGFP expression using CaMV 35S promoter and rbcS ployA as a terminator. The plasmid CP507-YFP (10.7 kb) containing yellow fluorescent protein uses CaMV 35S promoter to promote YFP expression and NOS as the terminator to form a YFP expression box (**Figures 6A** **, B**). The CP507-YFP plasmid was provided by the Quality Molecular Research Group of IVF-CAAS, and was propagated and preserved in our laboratory. The plasmid was amplified in the *E. coli* DH5α strain, and the EndoFree Maxi plasmid kit (TIANGEN Biotech Co., Ltd., Beijing, China) was used to extract the plasmid. The purified plasmid was stored at –20°C for later use.

Cellulase “onozuka” R-10 and pectolyase Y-23 were purchased from YAKULT HONSHA CO., LTD. Mannitol, bovine serum albumin (BSA), fluorescein diacetate (FDA), 2-(N-morpholino) ethanesulfonic acid (MES), polyethylene glycol 4000 (PEG 4000) and other reagents were purchased from Beijing Exelon Biotechnology Co., Ltd. The *E. coli* DH5α strain was purchased from Shanghai Weidi Biotechnology Co., Ltd.

### Cultivation of aseptic seedlings

Selected filled seeds were sterilized in 50 mL centrifuge tubes containing 75% alcohol for 3 min and then poured into 8% sodium hypochlorite for 8 min, rinsed three times with sterile water, and spread on seeding medium (4.45 g/L MS + 28 g/L sucrose + 8 g/L agar, pH 5.8). The cultures were incubated for 7-10 d at 25°C in low light (150 μmol m-2 s-1, 16 h light, 8 h dark).

### Protoplast isolation and purification

Upper fully expanded broccoli cotyledons of aseptic seedlings cultured for 7-10 d were selected (**Figure 5**), spread over the bottoms of sterile 9-cm glass Petri dishes filled with CM (**Table S1**), and incubated in the dark at 4°C for 12 h. After pretreatment, the CM was discarded, a scalpel was used to make a scratch approximately 1 mm long on the back of the leaf, and 10 mL of preconfigured enzyme digestion solution (0.5% cellulase R-10, 0.1% pectolyase Y-23, and 0.6 M mannitol, pH 5.8) was added. The material was placed on a shaker and enzymatically digested at 25°C in the dark for 3 h. After enzymatic digestion, the dishes were gently shaken in a single direction to release the protoplasts. The leaf residue was removed by passage of the material through a 50.0-μm nylon membrane sieve. The material that passed through the sieve was collected in a 10-mL round-bottom centrifuge tube. After centrifugation of this material at 700 r/min for 5 min, the supernatant was discarded, 7.0 mL of W5 (154 mM NaCl, 125.0 mM CaCl_2_, 5.0 mM KCl and 2.0 mM MES, pH 5.7) was added to the pellet, the resulting suspension was centrifuged at 700 r/min for 3 min, and the supernatant was again discarded. The above process was repeated twice. The protoplasts were then immediately resuspended in MMG solution (0.4 M mannitol, 15.0 mM MgCl_2_ and 4.0 mM MES, pH 5.7) at a density of 1.0-2.0×10^6^ protoplasts mL^-1^.

The yield of protoplasts was calculated using the hemocytometer counting method. The yield of obtained protoplasts (cells g^-1^) = N×10^4^×V×m^-1^, where N = the number of protoplasts counted in a hemocytometer chamber, V = the volume of diluted protoplasts, and m = the fresh weight of the cotyledons used for protoplast isolation. The viability of the protoplasts obtained was checked using fluorescein diacetate (FDA). Ten microliters of 0.01% fluorescein diacetate (FDA) were added to 500 microliters of protoplast suspension. After 5 min, the protoplasts were examined with an Olympus BX51 fluorescence microscope (green fluorescence, Olympus, Japan). The viability of the obtained protoplasts (%) = the number of protoplasts with green fluorescence/total protoplasts in the field (×100%). The experiment was repeated three times, and the average value was taken (n = 3).

The isolation and purification of broccoli cotyledon protoplasts of the three genotypes was performed using the above procedure.

### PEG-Ca^2+^-mediated protoplast transfection

The method referenced by Zhang et al. (2011) to transfect broccoli protoplasts was slightly optimized (Zhang et al., 2011). Plasmids (5-15 μg) were added to 200 μL of protoplast suspension (approximately 4×10^5^ protoplasts), followed by addition of an equal volume of PEG-Ca^2+^ solution (PEG4000, 0.2 M mannitol and 0.1 M CaCl_2_), and transformation was allowed to proceed in the dark at room temperature (approximately 25°C) for 20 min. Two volumes of W5 solution were then added to stop the reaction, the sample was centrifuged at 700 r/min for 3 min, the supernatant was discarded, and 1.0 mL of W5 solution was added to resuspend the pelleted material. The protoplasts were transferred to a 3.0-cm petri dish and cultured at 25°C in the dark for 12-15 h. The concentration of protoplasts used for transformation and the amount of W5 solution used to culture the transformants can be appropriately increased or reduced according to the purpose of the experiment. At the end of the culture period, the expression of fluorescent protein was observed under a fluorescence microscope, and the results were used to calculate the transformation efficiency of different materials.

Protoplast transformation efficiency was defined as the number of protoplasts with green fluorescence/total protoplasts in the field (×100%). The experiment was repeated three times, and the average value was taken (n = 3).

To obtain higher protoplast transformation efficiency, stepwise gradients of the factors affecting the transformation were tested: plasmid masses of 5, 10 and 15 μg and mass concentrations of PEG4000 in the PEG-Ca^2+^ solution of 20% (w/v) and 40% (w/v) were tested. The transformed materials were cotyledon protoplasts of different broccoli genotypes of B1, B40, and B42.

### Plasmids of PHG-*CRa*-eGFP, PHG-*029100*-eGFP and PHG-*031350*-eGFP

To construct the PHB-CRa plasmid, the full-length 4.6-kb *CRa* gene (derived from cabbage) cloned in our laboratory was ligated to the PHB plasmid after single digestion of the PHB plasmid with XhoI. Subsequently, PHB-CRa was digested with BamHI/XbaI, and DNA ligase (M0202M, NEB, MA, USA) was used to ligate eGFP to construct a PHG-*CRa*-eGFP expression vector (**Figure 6C**).

The *Bol029100* gene was amplified by PCR using the specific primers P1 (F: ATCCTCGAGCTGCAGG AGCTCATGGCACCCGCATGCAGCAGAATC; R: CCCTTGCTCACCATCACTAGTCCAATCAGCTTCTGTCTCAAGAAATC), and the *Bol031350* gene was amplified using the specific primers P2 (F: ATCCTCGAGCTGCAGGAGCTCATGGCACCCTCTTGCAGTCCAATC; R: CCCTTGCTCACCATCACTAGTCCCCCAAATATCTGAGAGGGAATAAC). The PCR fragment was recovered after gel electrophoresis and ligated to the digested plasmid PHG-eGFP to construct the PHG-*029100*-eGFP and PHG-*031350*-eGFP expression vectors (**Figures 6D** **, E**).

### Microscopy

Fluorescence microscopy (Olympus BX51, Japan) was used to observe and photograph the expression of green and red fluorescent proteins in broccoli protoplasts transfected with the PHG-eGFP, and CP507-YFP plasmids. The subcellular localization of the fluorescent proteins was observed through laser confocal microscopy (Olympus BX51, Japan). GFP was excited using a 488-nm laser line.

### Statistical analysis

All data were analyzed by analysis of variance (ANOVA) using SPSS Statistics version 19.0 (IBM^®^, Chicago, IL, USA). Student’s t-test or one-way analysis of variance (ANOVA), and Duncan’s multiple tests were used to assess significant differences (*p* < 0.05).

## Data availability statement

The original contributions presented in the study are included in the article/**Supplementary Material**. Further inquiries can be directed to the corresponding author.

## Author contributions

ZL: Conceptualization, Formal analysis, Project administration, Resources, Writing - review & editing, Validation. DY: Data curation, Formal analysis, Investigation, Methodology, Software, Writing - original draft, Writing - review & editing. YZ: Methodology, Validation, Visualization, Software, Investigation. YL and FH: Methodology, Review & editing. All authors contributed to the article and approved the submitted version.
