# Supplementary material for: A high-efficiency PEG-Ca2+-mediated transient transformation system for broccoli protoplasts
Source: Front Plant Sci. 2022 Dec 12;13:1081321. doi: 10.3389/fpls.2022.1081321 (PMC9790990; doi:10.3389/fpls.2022.1081321)
Supplement: Supplementary file 1 [file DataSheet_1.docx]

**Supplementary data**

The following are the Supplementary data to this article.

Table S1. CM medium detailed composition.

| **Chemicals** | **Concentration (mg/L)** |
| --- | --- |
| KNO_3_ | 190 |
| MgSO_4_ | 37 |
| CaCl_2_·2H_2_O | 44 |
| KH_2_PO_4_ | 17 |
| FeSO_4_·7H_2_O | 2.79 |
| Na_2_-EDTA | 3.73 |
| MnSO_4_·4H_2_O | 2.23 |
| H_3_BO_3_ | 0.62 |
| ZnSO_4_·7H_2_0 | 0.86 |
| NaMoO_4_·2H_2_O | 0.025 |
| CuSO_4_·5H_2_O | 0.0025 |
| KI | 0.083 |
| CoCl·6H_2_O | 0.0025 |
| Inositol | 10 |
| Nicotinic acid | 0.5 |
| Glycine | 0.2 |
| Lactium | 100 |
| pH | 5.7-5.8 |

Table S2. Yield and viability of cotyledon protoplasts of different broccoli genotypes.

| **Genotype** | **B1** | | **B40** | | **B42** | |
| --- | --- | --- | --- | --- | --- | --- |
|  | **Yield**  **(10^6^ protoplasts/g/mL)** | **Vitality**  **(%)** | **Yield**  **(10^6^ protoplasts/g/mL)** | **Vitality**  **(%)** | **Yield**  **(10^6^ protoplasts/g/mL)** | **Vitality**  **(%)** |
| 1 | 53.3 | 97.2 | 58.4 | 96.4 | 62.3 | 95.1 |
| 2 | 70.0 | 95.4 | 60.4 | 96.0 | 98.4 | 95.0 |
| 3 | 64.5 | 98.5 | 50.5 | 98.2 | 75.4 | 96.5 |

Table S3. Transfection efficiency of 5-15 μg PHG-eGFP plasmids in B1, B40 and B42 protoplasts.

| **Genotype** | **Plasmid concentration (μg/200 μL protoplasts)** | | |
| --- | --- | --- | --- |
|  | **5** | **10** | **15** |
|  | **Transfection efficiency (%)** | | |
| B1 | 58.6% 65.8% 59.8% | 59.5% 64.7% 65.0% | 59.4% 69.7% 73.1% |
| B40 | 21.4% 25.0% 15.5% | 34.5% 27.3% 17.6% | 27.4% 32.4% 29.4% |
| B42 | 13.0% 24.5% 12.8% | 32.2% 21.0% 10.4% | 30.4% 16.8% 28.2% |

Table S3. Transfection efficiency of 5-15 μg CP507-YFP plasmids in B1, B40 and B42 protoplasts.

| **Genotype** | **Plasmid concentration (μg/200 μL protoplasts)** | | |
| --- | --- | --- | --- |
|  | **5** | **10** | **15** |
|  | **Transfection efficiency (%)** | | |
| B1 | 42.2% 38.8% 44.8% | 44.5% 30.0% 55.2% | 48.8% 47.5% 38.2% |
| B40 | 25.4% 40.2% 41.8% | 35.0% 45.2% 53.0% | 44.2% 39.4% 38.8% |
| B42 | 15.4% 24.5% 18.8% | 24.2% 21.2% 27.4% | 33.3% 25.4% 20.0% |
